# Phase separation in mullite-composition glass

**DOI:** 10.1038/s41598-022-22557-7

**Published:** 2022-10-21

**Authors:** Stephen K. Wilke, Chris J. Benmore, Jan Ilavsky, Randall E. Youngman, Aram Rezikyan, Michael P. Carson, Vrishank Menon, Richard Weber

**Affiliations:** 1 Materials Development, Inc., Evanston, IL 60202 USA; 2 X-Ray Science Division, Advanced Photon Source, Argonne National Laboratory, Argonne, IL 60439 USA; 3 Science and Technology Division, Corning Incorporated, Corning, NY 14831 USA

**Keywords:** Phase transitions and critical phenomena, Structure of solids and liquids, Geochemistry, Geology

## Abstract

Aluminosilicates (AS) are ubiquitous in ceramics, geology, and planetary science, and their glassy forms underpin vital technologies used in displays, waveguides, and lasers. In spite of this, the nonequilibrium behavior of the prototypical AS compound, mullite (40SiO_2_-60Al_2_O_3_, or AS60), is not well understood. By deeply supercooling mullite-composition liquid via aerodynamic levitation, we observe metastable liquid–liquid unmixing that yields a transparent two-phase glass, comprising a nanoscale mixture of AS7 and AS62. Extrapolations from X-ray scattering measurements show the AS7 phase is similar to vitreous SiO_2_ with a few Al species substituted for Si. The AS62 phase is built from a highly polymerized network of 4-, 5-, and 6-coordinated AlO_x_ polyhedra. Polymerization of the AS62 network and the composite morphology provide essential mechanisms for toughening the glass.

## Introduction

Mullite is an eminent material throughout the field of ceramics, from pottery and porcelains to refractories and thermal barrier coatings^1^. As a phase in the CaO-MgO–Al_2_O_3_-SiO_2_ (CMAS) system, which is present throughout the Universe, it also represents an important geological material, formed at the Earth’s surface when basaltic magmas contact clay minerals^2^. In the aluminosilicate (AS) binary, the mullite composition (40SiO_2_-60Al_2_O_3_, or AS60) is effectively an endmember of AS-based glasses. These constitute a large fraction of functional glasses due to their hardness and toughness^3^. A key to obtaining these glasses’ desirable properties is navigating around the system’s metastable liquid–liquid immiscibility during processing, which can lead to phase-separated glasses in the SiO_2_-rich region of the phase diagram upon melt quenching^4^. Avoiding or manipulating this immiscibility provides the means to control properties of the resulting glasses.

**Figure 1:**
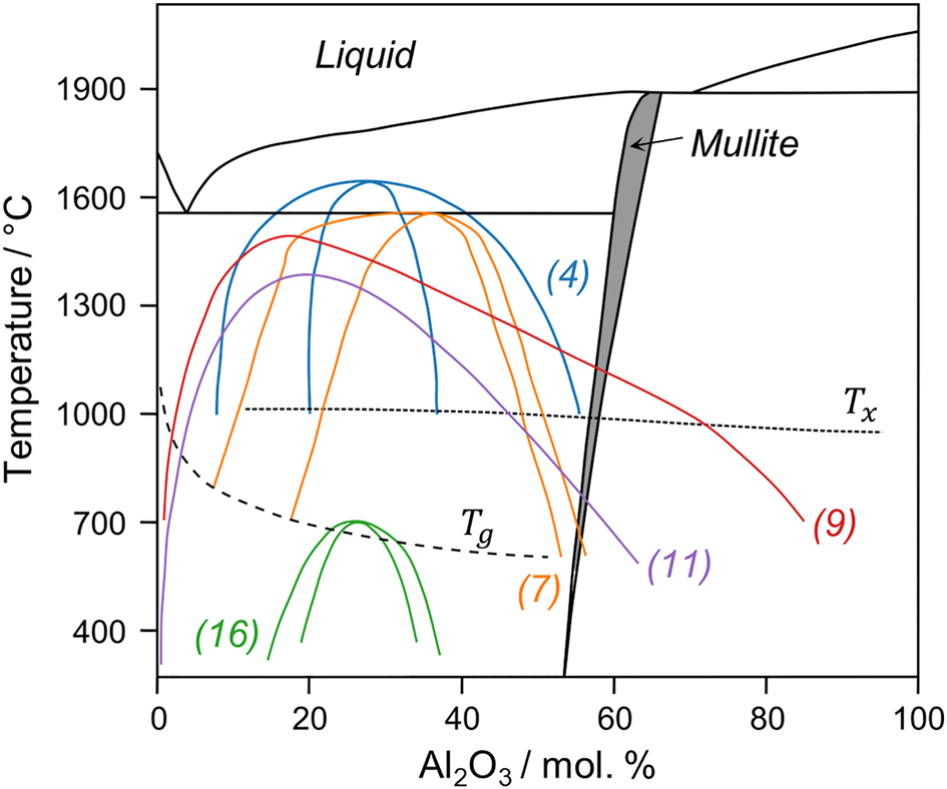
Metastable liquid–liquid immiscibility in SiO_2_-Al_2_O_3_. Numerous locations for the miscibility gap have been proposed based on experimental observations of quenched glasses and thermodynamic calculations (colored curves with Ref. numbers given in figure). The compositional and temperature limits of immiscibility vary substantially between studies, with the Al_2_O_3_-rich endmember intersecting ${T}_{g}$ anywhere from 56 to > 85 mol. %. Equilibrium phase diagram adapted from Mao et al.^11^. Glass transition is ${T}_{g}$^7^, and crystallization of glasses upon heating is ${T}_{x}$^41^.

The known AS miscibility gap ranges qualitatively from near SiO_2_ and ends prior to the mullite composition, approximately AS7-AS56. Yet, substantial disagreement persists regarding the compositional and temperature limits of immiscibility (Fig. 1). Many experimental studies have located the mullite composition outside the miscibility gap^4–6^, while different thermodynamic models have predicted it within^7,8^ or outside^9–11^ the gap (see Fig. 1). In this regard, though mullite is ubiquitous in ceramics, its structural role in AS phase separation remains an unsolved mystery. This mainly arises because of the high temperatures involved and the metastable nature of the liquid–liquid phase separation dome, which exists hundreds of degrees below the equilibrium melting point (${T}_{m}$ ~ 1890 °C for mullite^11^). Rosales-Sosa et al*.*^12^ recently reported a mullite-composition glass with exceptional hardness (8.07 GPa) and crack resistance (55.4 N), which sparks renewed curiosity about the structure of mullite-composition glass and an explanation for these desirable properties. (Hereafter, mullite-composition glass is referred to as “mullite glass,” defined as the glassy form obtained by melt quenching of the AS60 liquid.) Here, we find that mullite glass is in fact two-phase, with nanometer domains of SiO_2_-rich glass embedded in a glassy, polymerized Al_2_O_3_-rich network. Glass structure and electron microscopy measurements provide unambiguous evidence of liquid–liquid phase separation in mullite and provide an experimentally-based estimate for the high-Al_2_O_3_ limit of AS immiscibility. Atomic structure modeling of this Al-rich endmember illuminates the structural underpinnings of mullite glass’s excellent crack resistance.

## Results and discussion

**Figure 2:**
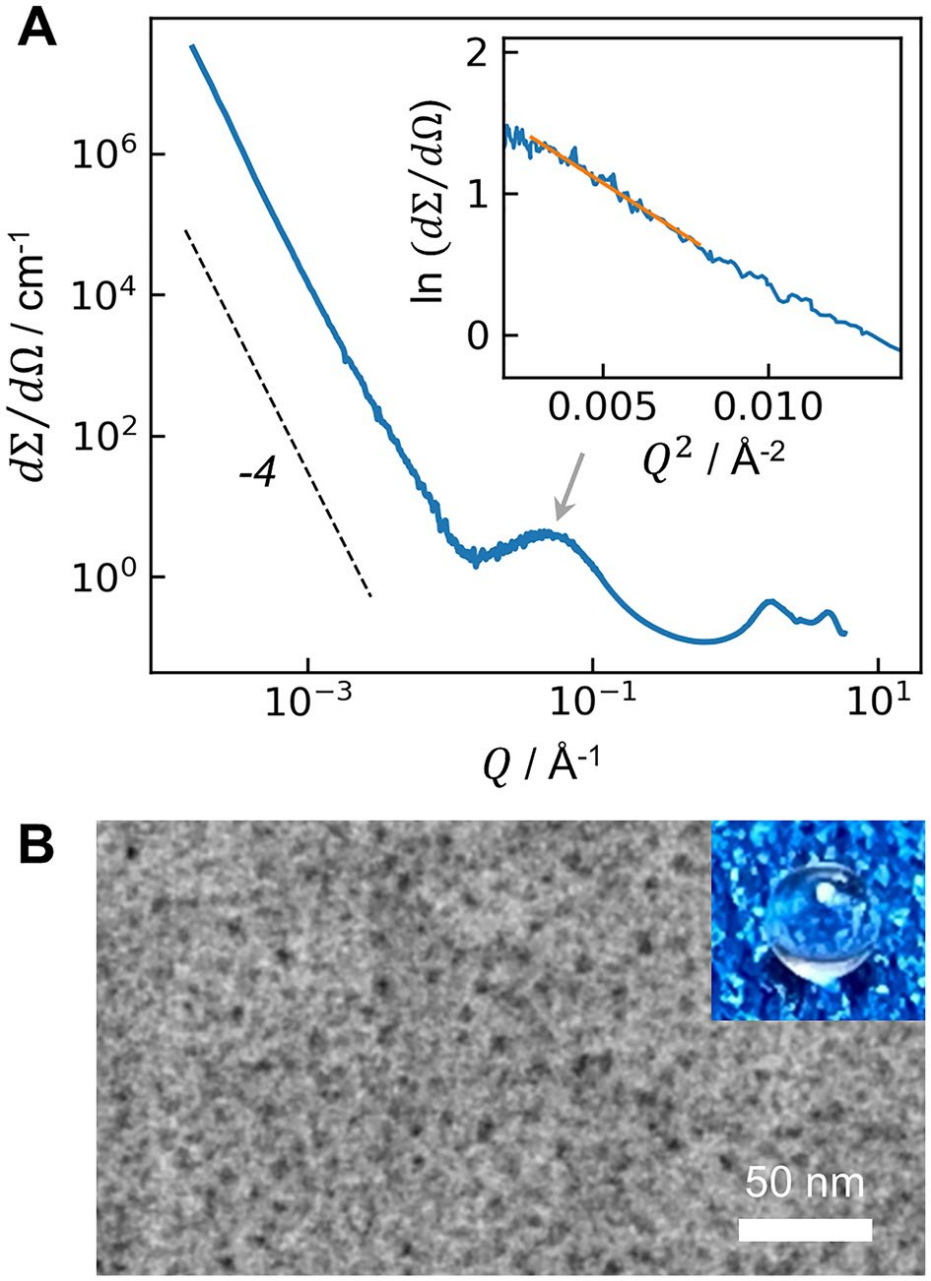
Phase separation in mullite-composition glass. (**A**) X-ray scattering differential cross section exhibits a Porod slope to the right of a small-angle peak near 0.05 Å^−1^ (gray arrow), indicative of phase-separated domains. Inset: Guinier analysis predicts an average domain size of 5.5 nm. (**B**) High-angle annular dark field STEM image of a 40–100 nm thick mullite-composition glass specimen, revealing contrast between the 4–6 nm Si-rich domains (black) and surrounding Al-rich matrix. Inset: transparent glass bead, 1.3 mm in diameter.

Because of its reticence to vitrify, mullite-composition glasses are often partially crystalline^13,14^, and fully glassy materials are typically limited to particle sizes of 10s or 100s of microns, prepared via roller quenching or other techniques that achieve extremely fast cooling (~ 10^6^ °C s^−1^)^1^. Here, fully glassy beads, approximately 2 mm in diameter, were prepared using aerodynamic levitation and laser beam heating^15^, which avoids heterogeneous nucleation while maintaining moderate cooling rates ($\le$ 10^3^ °C s^−1^). This is crucial because the fast rates associated with roller quenching have previously obfuscated the full extent of AS phase separation^16,17^. Phase separation in the glass is evident from ultra-small and small-angle X-ray scattering (Fig. 2A), which exhibits a peak near $Q$ = 0.05 Å^−1^, where $Q$ is the momentum transfer given by $Q=4\pi \mathrm{sin}(\theta )/\lambda$ and 2 $\theta$ is the scattering angle. Using Guinier analysis (Fig. 2A inset) and assuming a spherical scatterer, this peak corresponds to domains with a diameter of 5.5 nm. High-angle annular dark field scanning transmission electron microscopy (STEM, Fig. 2B) reveals separated Si-rich domains within an Al-rich matrix, with a domain size of 4–6 nm consistent with the Guinier analysis. From the apparent areal fractions in the STEM image and assuming a Si-rich endmember composition of AS7, based on previous studies^7,18,19^, the Al-rich endmember phase has a composition of 61.9(7) mol% Al_2_O_3_ (~ AS62). This AS62 estimate significantly extends the experimentally-observed immiscibility range beyond the AS56 limit suggested by previous direct observations^4,5^, and it coincides with the intersection of ${T}_{g}$ with the miscibility gap proposed by Mao et al.^11^ in their thermodynamic reassessment of the SiO_2_-Al_2_O_3_ system (Fig. 1, purple curve). Despite the phase separation, the mullite glass is optically transparent (Fig. 2B, inset).

According to an exhaustive review of phase separation behavior in binary silicate systems by Hudon and Baker^20^, liquid–liquid separation in aluminosilicates is driven by coulombic repulsions between poorly screened Al^3+^ cations. In these glasses, Al^3+^ is an amphoteric cation, referring to its ability to adopt different coordination numbers with oxygen: 4, 5, and 6. The partial covalent character of tetrahedral AlO_4_ species reduces their electrostatic repulsions with other cations, but the Al^3+^ in octahedral AlO_6_ species are poorly screened. To achieve stoichiometric charge balance in the AS binary system, both AlO_4_ and AlO_6_ coexist, thus leading to phase separation.

**Figure 3:**
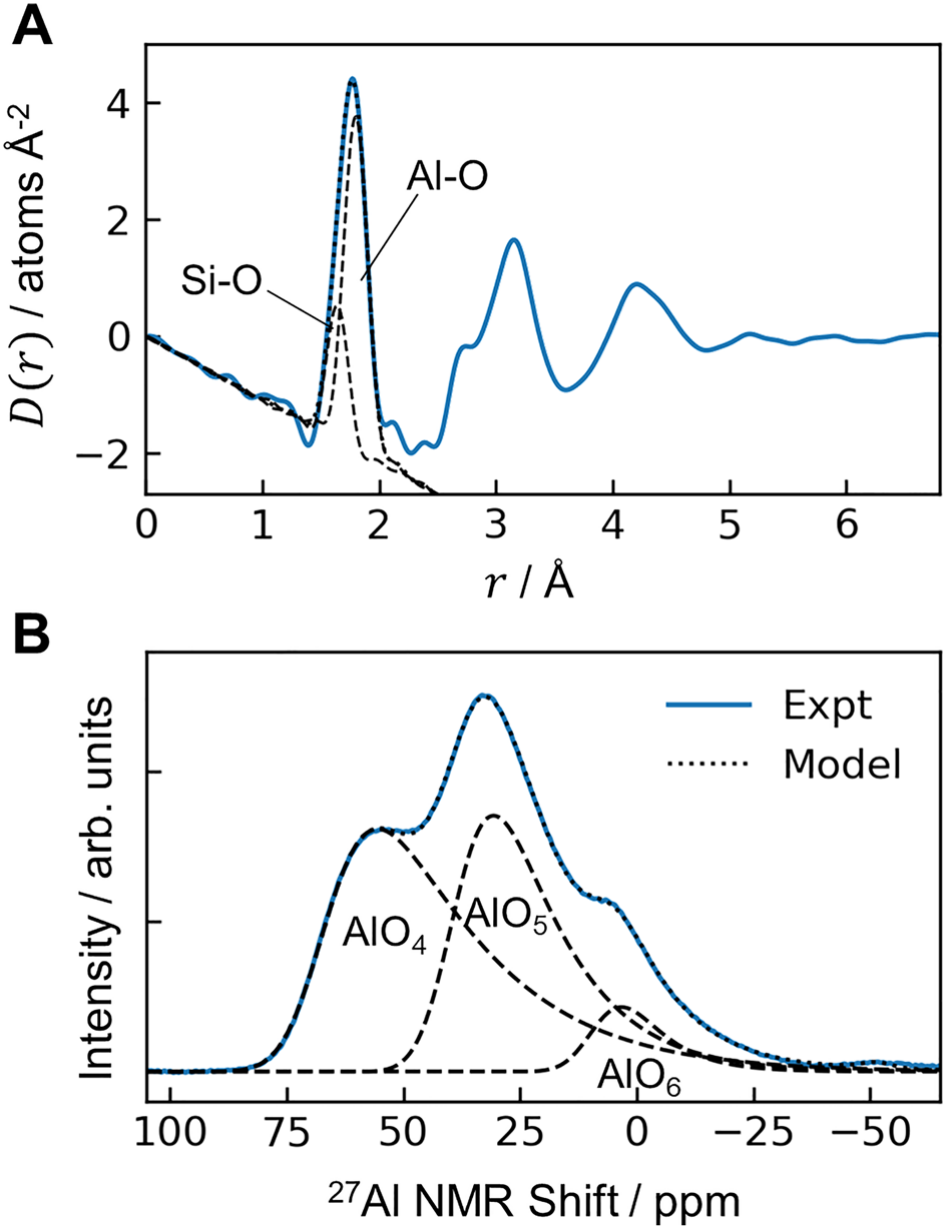
Average local atomic structure in mullite-composition glass. (**A**) Differential PDF from high-energy X-ray diffraction. The slope for $r$ < 1 Å is $-4\pi \rho$, with $\rho$ = 2.912 g cm^−3^, or 0.08640 atoms Å^−3^. Gaussian functions were fit for the weighted Si–O and Al-O partial pair correlations. (**B**) The ^27^Al MAS NMR spectrum exhibits three peaks corresponding to different Al speciation.

The local atomic structure in mullite glass was probed with high-energy X-ray diffraction and ^27^Al magic angle spinning nuclear magnetic resonance (MAS NMR) spectroscopy. For X-ray diffraction, the total structure factor, $S(Q)$, is Fourier transformed to obtain the real-space differential pair distribution function (PDF), $D(r)$, shown in Fig. 3A (see Supplementary Information S1 (SI) for PDF definitions). The first PDF peak near 1.77 Å comprises overlapping contributions from the Si–O and Al-O atomic partial pair correlations. Fitting Gaussian functions to these peaks and integrating the Al-O peak yields the mean atomic coordination, ${n}_{AlO}$ = 4.38(7). The glass lacks significant local order beyond the second (Si/Al)-O coordination shell, i.e., above 5 Å. The ^27^Al MAS NMR spectrum (Fig. 3B) contains three overlapping peaks at 67.9, 40.4, and 9.9 ppm, corresponding to AlO_4_, AlO_5_, and AlO_6_ species, respectively^14^. Using peak shapes guided by separate ^27^Al triple-quantum MAS (3QMAS) NMR measurements on the same glass (SI, Fig. S1), integration of these peaks yields relative population fractions of 0.498(42), 0.430(41), and 0.072(10), respectively, and a mean coordination of ${n}_{AlO}$ = 4.57(27) consistent with the X-ray analysis. The presence of AlO_5_ and AlO_6_ species is also consistent with reports of Raman spectroscopy for Al_2_O_3_-rich silicate glasses^13,21^. The substantial AlO_5_ fraction, absent in crystalline mullite^22^, is a structural motif shared in common with the liquid^23^ that is quenched into the glass structure^6^.

**Figure 4:**
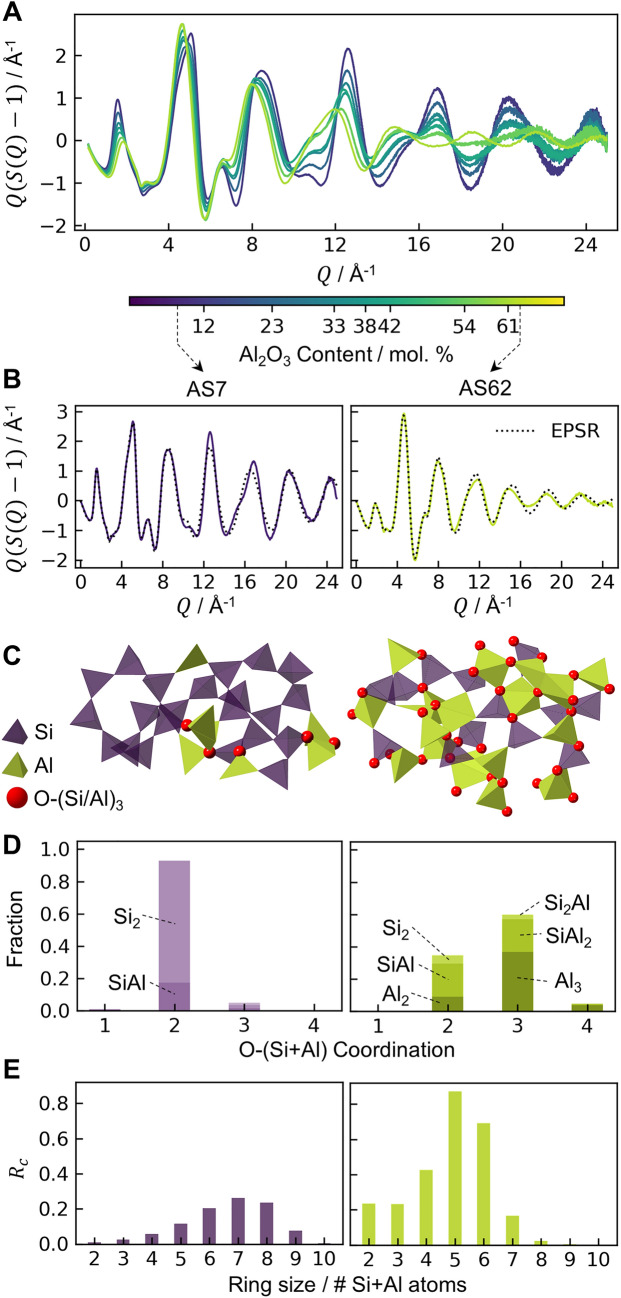
Structure of the endmembers in phase-separated mullite and aluminosilicate glasses. (**A**) X-ray diffraction interference functions of phase-separated glasses, with compositions ranging AS12-AS61. (**B**) Interference functions for the endmember compositions, AS7 and AS62, linearly extrapolated from the glasses in (A). EPSR provides structural models of the endmembers consistent with the experimental data: (**C**) structure visualizations, (**D**) oxygen coordination environments, and (**E**) ring size distributions of AS7 and AS62 endmembers. ${R}_{c}$ is the number of -O-(Si/Al)- rings normalized by the number of atoms in the simulation volume.

To gain further structural insights, experimentally-constrained models for the phase-separated endmembers are sought. X-ray diffraction interference functions, $Q(S\left(Q\right)-1)$, were measured for compositions AS12-AS61 (Fig. 4A), spanning the immiscibility range. These glasses are each phase-separated into the same endmembers, whose relative fractions vary linearly with glass composition. The interference functions were therefore extrapolated^24^ to predict the interference functions for the AS7 and AS62 endmembers (Fig. 4B), for which atomistic models were obtained by Empirical Potential Structure Refinement (EPSR)^25^.

The structure of AS7 (Fig. 4C) is similar to that of SiO_2_ glass: O atoms are 93% bridging (i.e., connecting two (Si/Al)O_x_ polyhedra), 5% form triclusters^26^ (Fig. 4D), and the network comprises 99% corner-sharing between polyhedra that form rings with a distribution modal size of 7 cations (Fig. 4E). In contrast, the AS62 structure contains 60% O as triclusters, making it much more topologically rigid^27^. Oxygen triclusters are a charge balancing mechanism^20^ and here are associated with Al-rich environments such as O-Al_3_ and O-SiAl_2_. While substantial, this large tricluster fraction is consistent with molecular dynamics (MD) simulations for mullite glass^28^ and is reasonable in comparison to MD predictions of 82% triclusters in melt quenched Al_2_O_3_^29^. The AS62 network contains a mixture of AlO_x_ species: 57% 4-, 37% 5-, and 6% 6-coordinate. These polyhedra and the SiO_4_ are connected via 86% corner- and 13% edge-sharing, forming more than 3 $\times$ the number of rings as compared to AS7. This large number of rings makes AS62 highly polymerized.

Nanoscale phase separation and atomic structure are the keys to understanding AS glasses’ trend of increasing hardness—and, anomalously, crack resistance—with Al_2_O_3_ content^12^. For a single phase, hardness is proportional to density^12^, so the higher density of AS62 leads to higher hardness compared to AS7. For any glass composition within the miscibility gap, the glass comprises domains of the two endmember phases, AS7 and AS62, with relative volume fractions that are linearly dependent on composition. This linear dependence of the volume fractions explains the linear relationship of hardness with increasing Al_2_O_3_ content. This trend also matches the linear increase of O triclusters (SI, Fig. S2A), which topologically harden the atomic network. Cracking resistance (CR) in glass is often attributed to the availability of free volume to accommodate plastic deformation, so CR typically decreases with density, yet the opposite is observed for the AS compositional series. Rosales-Sosa et al*.* hypothesized this is due to rearrangement of the multiple AlO_x_ environments, enabling shear deformation^12^, which is supported by the low energy barrier predicted by MD for AlO_5_ configurational transitions in AS glasses^30^.

The discovery of phase separation in mullite glass and the structure of the Al-rich endmember point to two additional CR mechanisms. First, the highly polymerized network in AS62 makes it more difficult to form cavities that lead to crack formation, according to MD simulations of high- and low-brittleness single-phase AS glasses under mechanical loading^31^. These simulations also showed that cracking is mitigated by the breaking of larger rings (containing ~ 6 cations) into 3- and 4-member rings, so the much larger population of rings in AS62 is supportive of its higher CR compared to AS7. Second, CR has been observed to increase nonlinearly with Al_2_O_3_ content: from 8 to 20 N for AS30 to AS55, then jumping to 55 N for AS60^12^. This jump can be explained by the relative fractions and morphologies of the phase-separated domains. As Al_2_O_3_ content increases from AS55 to AS60, the more easily-cracked AS7 phase becomes sufficiently disconnected (Fig. 2B) such that cracks can no longer propagate continuously through AS7, so the more crack-resistant AS62 matrix thus suddenly increases CR.

Commercial glasses can be toughened by surface modification (e.g., ion exchange^32^) and/or by creating a composite structure that results in crack deflection (e.g., in glass-ceramics^33^). This work shows the potential for using phase-separated vitreous materials. The presence of a highly polymerized network and the composite nature of the two-phase glass offer additional design approaches in the toolbox for glass strengthening. Combination of techniques, for example, selective ion exchange of only one phase in a separated glass, may open up new possibilities. Though mullite is a reluctant glass former, its ~ 390 °C difference between ${T}_{g}$ and ${T}_{x}$ (Fig. 1) suggests it could be formed in thin sheets, where cooling rates are sufficiently high and heterogeneous nucleation can be avoided.

## Methods

Full information on the experimental methods is provided in the SI.

Glass beads were prepared from mixtures of SiO_2_ and Al_6_Si_2_O_13_ powders using aerodynamic levitation and heating with a 10.6 μm CO_2_ laser beam^15^. Nominal compositions of 10, 20, 30, 35, 40, 50, and 60 mol. % Al_2_O_3_ (AS10-AS60) were heated to 2000 °C and then cooled at rates < 10^3^ °C s^−1^ to obtain colorless, transparent glass beads 1.5–2 mm in diameter. Evaporation of SiO_2_ caused 2–10% mass loss during melt processing, resulting in final compositions consistent with energy dispersive spectroscopy on polished cross-sections: AS12, 23, 33, 38, 42, 54, and 61. For the mullite glass (AS61), annular dark field STEM images were collected at 200 keV beam energy. Specimens were prepared by in situ lift-out from a glass cross-section, followed by ion beam thinning at 5 keV. The composition of the Al_2_O_3_-rich endmember was estimated to be 61.9(7) mol. % Al_2_O_3_, or ~ AS62, from the areal fractions observed in STEM, assuming a SiO_2_-rich endmember composition of AS7^7,18,19^.

^27^Al MAS NMR spectroscopy was conducted at an external field strength of 16.4 T, spinning at 22 kHz, and referenced to an external shift standard of aqueous aluminum nitrate at 0.0 ppm. Data were processed with VnmrJ and DMFit softwares, using the Czjzek function to represent each of the three resonances^34^. The quadrupolar coupling constant, isotropic chemical shift, and peak areas were extracted from peak fits^35^. ^27^Al 3QMAS NMR data were collected using standard two-pulse experiments with a z-filter, and analyses of these data were used to further guide fitting of the ^27^Al MAS NMR data.

X-ray scattering measurements were collected for the ultra-small and small-angle range with 21 keV X-rays^36^, as well as the small- and wide-angle range with 60 keV X-rays^37^. For PDF analysis, dedicated wide-angle scattering (100 keV) data were reduced to obtain the total structure factors and then Fourier transformed to obtain the PDFs^38^. Mean bond distances and coordinations for Si–O and Al-O were extracted from the PDFs with Gaussian peak fits^39^. Structure factors for glasses in the AS12-AS42 compositional range were extrapolated to estimate the structure factors for the AS7 and AS62 phase-separated endmembers. Structural models for AS7 and AS62 were created using EPSR^25^, a Monte Carlo based technique that perturbs a system with known composition, density, and simple interatomic potentials, to optimize agreement between the experimental and simulated scattering. Atomic coordination distributions and ring statistics^40^ were calculated from these models.

## Supplementary Information

Supplementary Information 1.

Supplementary Information 2.

## Data Availability

All data are available upon reasonable request from the corresponding author. Structure factor data are provided in the SI for the X-ray diffraction measurements.

## References

[1] Schneider, H. & Komarneni, S. *Mullite* (Wiley-VCH, 2005).

[2] Bowen NL, Greig JW, Zies EG (1924). Mullite, a silicate of alumina. J. Washingt. Acad. Sci..

[3] Kohli JT, Hubert M, Youngman RE, Morse DL (2022). A Corning perspective on the future of technical glass in our evolving world. Int. J. Appl. Glas. Sci..

[4] MacDowell JF, Beall GH (1969). Immiscibility and crystallization in Al2O3-SiO2 glasses. J. Am. Ceram. Soc..

[5] Risbud SH, Pask JA (1978). Mullite crystallization from SiO2-Al2O3 melts. J. Am. Ceram. Soc..

[6] Poe BT, McMillan PF, Angell CA, Sato RK (1992). Al and Si coordination in SiO2-Al2O3 glasses and liquids: A study by NMR and IR spectroscopy and MD simulations. Chem. Geol..

[7] Risbud SH, Pask JA (1977). Calculated thermodynamic data and metastable immiscibility in the system SiO_2_-Al2O_3_. J. Am. Ceram. Soc..

[8] Djuric M, Mihajlov A, Petrasinovic-Stojkanovic L, Zivanovic B (1996). Thermodynamic analysis of the metastable regions for the Al2O3-SiO2 system. J. Am. Ceram. Soc..

[9] Ban T, Hayashi S, Yasumori A, Okada K (1996). Calculation of metastable immiscibility region in the Al2O3–SiO2 system. J. Mater. Res..

[10] Takei T, Kameshima Y, Yasumori A, Okada K (2000). Calculation of metastable immiscibility region in the Al2O3-SiO2 system using molecular dynamics simulation. J. Mater. Res..

[11] Mao H, Selleby M, Sundman B (2005). Phase equilibria and thermodynamics in the Al2O3-SiO2 system—Modeling of mullite and liquid. J. Am. Ceram. Soc..

[12] Rosales-Sosa GA, Masuno A, Higo Y, Inoue H (2016). Crack-resistant Al2O3-SiO2 glasses. Sci. Rep..

[13] Okuno M, Zotov N, Schmücker M, Schneider H (2005). Structure of SiO2–Al2O3 glasses: Combined X-ray diffraction, IR and Raman studies. J. Non. Cryst. Solids.

[14] Weber R, Sen S, Youngman RE, Hart RT, Benmore CJ (2008). Structure of high alumina content Al2O3-SiO2 composition glasses. J. Phys. Chem. B.

[15] Weber JKR (2010). The containerless synthesis of glass. Int. J. Appl. Glas. Sci..

[16] Jantzen C, Schwahn D, Schelten J (1978). Phase Decomposition in Al2O3-SiO2 Glasses. J. Appl. Cryst..

[17] Risbud SH, Pask JA (1978). On the location of metastable immiscibility in the system SiO2-Al2O3. J. Am. Ceram. Soc..

[18] Nassau K, Shiever JW, Krause JT (1975). Preparation and properties of fused silica containing alumina. J. Am. Ceram. Soc..

[19] Sen S, Youngman RE (2004). High-resolution multinuclear NMR structural study of binary aluminosilicate and other related glasses. J. Phys. Chem. B.

[20] Hudon, P. & Baker, D. R. The nature of phase separation in binary oxide melts and glasses. I. Silicate systems. *J. Non. Cryst. Solids* **303**, 299–345 (2002).

[21] McMillan P, Piriou B (1982). The structures and vibrational spectra of crystals and glasses in the silica-alumina system. J. Non. Cryst. Solids.

[22] Rehak P (1998). Study of the Al coordination in mullites with varying Al: Si ratio by 27Al NMR spectroscopy and X-ray diffraction. Am. Mineral..

[23] Krishnan S, Weber JRK, Ansell S, Hixson AD, Nordine PC (2000). Structure of Liquid Al6Si2O13 (3:2 Mullite). J. Am. Ceram. Soc..

[24] Soper, A. K., & Ricci, M. A. Structures of high-density and low-density water. *Phys. Rev. Lett.* **84**, 2881 (2000). 10.1103/PhysRevLett.84.2881 11018966

[25] Soper AK (1996). Empirical potential Monte Carlo simulation of fluid structure. Chem. Phys..

[26] Stebbins JF, Xu Z (1997). NMR evidence for excess non-bridging oxygen in an aluminosilicate glass. Nature.

[27] Welch RS (2021). Topological hardening through oxygen triclusters in calcium aluminosilicate glasses. J. Am. Ceram. Soc..

[28] Liao K (2020). Real-space mapping of oxygen coordination in phase-separated aluminosilicate glass: Implication for glass stability. ACS Appl. Nano Mater..

[29] Shi C (2019). The structure of amorphous and deeply supercooled liquid alumina. Front. Mater..

[30] Wiles NT, Goyal S, Baker SP (2020). Geometric configuration of five-coordinated Al and Si in tectosilicate calcium aluminosilicate glasses and its effect on plastic flow. J. Non. Cryst. Solids.

[31] Ito S (2004). Structural study on mechanical behavior of glass. J. Ceram. Soc. Japan.

[32] Beall GH (2016). Ion-exchange in glass-ceramics. Front. Mater..

[33] Okuma G, Maeda K, Yoshida S, Takeuchi A, Wakai F (2022). Morphology of subsurface cracks induced by Vickers indentation observed by synchrotron X-ray multiscale tomography. Sci. Rep..

[34] Massiot D (2002). Modelling one- and two-dimensional solid-state NMR spectra. Magn. Reson. Chem..

[35] Neuville DR, Cormier L, Massiot D (2004). Al environment in tectosilicate and peraluminous glasses: A 27Al MQ-MAS NMR, Raman, and XANES investigation. Geochim. Cosmochim. Acta.

[36] Ilavsky J (2018). Development of combined microstructure and structure characterization facility for in situ and operando studies at the Advanced Photon Source. J. Appl. Crystallogr..

[37] Benmore, C. J. *et al.* Extended range X-ray pair distribution functions. *Nucl. Instruments Methods Phys. Res. Sect. A Accel. Spectrometers, Detect. Assoc. Equip.* **955**, 163318 (2020).

[38] Benmore CJ (2012). A review of high-energy X-ray diffraction from glasses and liquids. ISRN Mater. Sci..

[39] Pickup D, Moss R, Newport R (2014). NXFit: A program for simultaneously fitting X-ray and neutron diffraction pair-distribution functions to provide optimized structural parameters. J. Appl. Crystallogr..

[40] Le Roux S, Jund P (2010). Ring statistics analysis of topological networks: New approach and application to amorphous GeS2 and SiO2 systems. Comput. Mater. Sci..

[41] Takamori T, Roy R (1973). Rapid Crystallization of SiO2-Al2O3 glasses. J. Am. Ceram. Soc..

