# Supplementary material for: Phase separation in mullite-composition glass
Source: Sci Rep. 2022 Oct 21;12:17687. doi: 10.1038/s41598-022-22557-7 (PMC9587060; doi:10.1038/s41598-022-22557-7)
Supplement: Supplementary file 1 — Supplementary Information 1. [file 41598_2022_22557_MOESM1_ESM.pdf]

Supplementary Information for:

Phase separation in mullite-composition glass

Stephen K. Wilke, Chris J. Benmore, Jan Ilavsky, Randall E. Youngman, Aram Rezikyan, Michael P. Carson, Vrishank Menon, Richard Weber

Materials and Methods

Glass Preparation and Composition

Glass beads were prepared with compositions across the aluminosilicate binary, nominally 10, 20, 30, 35, 40, 50, and 60 mol. % Al_2O_3 (AS10-AS60). Containerless processing[1] was used to maintain high sample purity, avoid heterogeneous nucleation during melt quenching, and enable vitrification at moderate cooling rates (i.e., < 10^3 °C s^{-1}). For each composition, powders of SiO_2 (99.5%, -325 mesh, Sigma-Aldrich) and mullite (99.9% $Al_6Si_2O_{13}$, Kyoritsu) were dried at 600 °C for 3 h, weighed, and mechanically mixed. Portions of each powder mixture were fused into spheroids in a Cu hearth using a 10.6 μm CO_2 laser. The spheroids were then levitated on an O_2 gas stream and again melted with the laser. The sample temperature, measured by an optical pyrometer (λ = 0.9 μm), was held near 2000 °C for 30 s before turning off the laser to cool the sample. Colorless, transparent glasses formed for sufficiently small samples, 1.5-2 mm diameter.

Evaporation of SiO_2 caused 2-10% mass loss during melt processing, and mass loss decreased with Al_2O_3 content of the starting powder.

Electron Microscopy

The compositions of glass beads were verified using energy dispersive spectroscopy (EDS) of ceramographically prepared cross-sections. Beads were mounted in acrylic, ground and polished to a final step with 1 μm diamond suspension, and sputter coated with Au/Pd. EDS measurements (Hitachi SU8030) were made on 4 sites across each bead cross-section, and compositions were in agreement with the calculated values based on SiO_2 mass loss: AS12, 23, 33, 38, 42, 54, and 61.

For the mullite-composition glass (AS61, "mullite glass"), annular dark field scanning transmission electron microscopy (STEM, aberration-corrected FEI Titan ChemiSTEM) images were collected at 200 keV beam energy. Specimens were prepared by *in situ* lift-out from a glass cross-section, using an Omniprobe needle inside a Quanta 3D 600 dual beam FIB/SEM instrument operated at 30 keV ion beam energy. The resulting glass lamella was thinned at 5 keV ion beam energy.

The composition of the Al_2O_3-rich endmember in the phase-separated mullite glass was estimated from the areal fractions observed in STEM, assuming a SiO_2-rich endmember composition of AS7, based on previous studies[2–4]. Due to depth averaging of the electron probe through the 40-100 nm thick specimen, the true volume fraction will be smaller than the observed areal fraction. To determine the ratio between volume and areal fractions, we consider a probed volume of dimensions $x \times y \times z$ containing N spheres of AS7 with radius R. The volume fraction is given by:

$$V_f = \left(N\frac{4\pi R^3}{3}\right)/xyz \qquad \text{(S1)}$$

Assuming no overlap of the spheres along the probe direction, z, the areal fraction is given by:

$$A_f = (N\pi R^2)/xy \qquad \text{(S2)}$$

and the ratio is

$$V_f/A_f = 4R/3z \qquad \text{(S3)}$$

Based on the domain size of $2R$ = 5.5 nm (from SAXS analysis) and STEM specimen thickness of 40-100 nm, this ratio would be 1/12 to 1/30. However, the mottled appearance of the STEM image (Fig. 2B) suggests considerable overlap of SiO_2-rich domains along the probe direction, which would lower the areal fraction. Two extremes of potential overlap are considered: substantial overlap, defined as 4 domains overlapping along the 40 nm of the probe direction, or minimal overlap, corresponding to 2 domains overlapping along 100 nm. This effect changes the possible range of the V_f/A_f ratio to 1/3 – 1/15.

The STEM image (Fig. 2B) shows a 0.085(25) areal fraction of AS7. Using the uncertainty for V_f/A_f, this corresponds to a mole fraction of 0.016(13) and an Al_2O_3-rich endmember composition of 61.9(7) mol. % Al_2O_3, or ~AS62.

Nuclear Magnetic Resonance Spectroscopy

^{27}Al NMR spectroscopy was conducted at an external field strength of 16.4 T using an Agilent DD2 spectrometer and 3.2 mm MAS NMR probe. Two glass beads were placed in a 3.2 mm zirconia rotor having low Al background, and the sample was spun at 22 kHz using compressed nitrogen. MAS NMR spectra were obtained with a single-pulse experiment, incorporating a radio-frequency (rf) pulse width of 0.6 μs (~ π/12 tip angle), a recycle delay of 5 s, and averaging of 8000 acquisitions. MAS NMR data were processed with commercial software (VnmrJ), without apodization and referenced to an external shift standard of aqueous aluminum nitrate at 0.0 ppm. DMFit was used to fit these data, using the Czjzek function to represent each of the three resonances[5]. The Gaussian Isotropic Model version of the Czjzek distribution describes each resonance with the peak value of the quadrupolar coupling constant, the isotropic chemical shift and its distribution, and peak areas[6]. Fitting results (Table S1) were compared with peak parameter estimates from ^{27}Al 3QMAS NMR spectroscopy, including relative magnitude of C_Q among the three different peaks. Results are consistent with treatment of similar data for other materials[7].

^{27}Al triple-quantum MAS (3QMAS) NMR data were collected using the standard two-pulse experiment with a z-filter (Fig. S1A). Calibrated rf pulses of 3.2 and 1.2 μs were used to excite and detect multiple quantum coherence, and a z-filter pulse width of 15 μs followed a delay of 45.45 μs (single rotor cycle). 2400 acquisitions were collected at each of 60 t_1 increments. ^{27}Al 3QMAS NMR data were processed in VnmrJ, including shear transformation, with 100 Hz line broadening in the MAS NMR dimension. 3QMAS NMR data were analyzed by using the peak positions (center of gravity) in both dimensions to estimate isotropic chemical shift and quadrupolar coupling product (P_Q), as outlined by Amoureux *et al.*[8]. Additional insight into the NMR parameters defining each of the lineshapes was obtained by taking MAS NMR slices through each peak maximum, and then fitting in DMFit with the Czjzek function (Fig. S1B), as outlined above for the ^{27}Al MAS NMR data. Site parameters from these 3QMAS NMR analyses were used to further guide fitting of the ^{27}Al MAS NMR data, which, although containing less resolution between the different Al resonances, is much more quantitative than multiple-quantum MAS methods.

X-ray Scattering

X-ray scattering measurements were performed at the Advanced Photon Source, Argonne National Laboratory (Lemont, IL). For mullite glass, ultra-small and small-angle scattering (Fig. 2A, $10^{-4} < Q < 5.9$ Å^{-1}) were measured at Sector 9-ID-C[9]. Glass beads were mounted with Scotch Magic Tape (3M), scattering of 21 keV X-rays was measured in a transmission geometry, and data were reduced with Nika software[10] and de-smeared to remove instrumental slit smearing.

For all AS glasses, phase separation was verified using combined small- and wide-angle scattering ($0.08 < Q < 16$ Å^{-1}) at Sector 6-ID-D (Fig. S3). Glass beads were mounted on Kapton tape, the diffracted intensity of 60 keV X-rays was detected with two Dexela 2315-MAM area detectors, and the datasets were spliced together after appropriate background subtraction and scaling[11].

For pair distribution function (PDF) analysis of atomic structure, wide-angle scattering measurements were collected at Sector 6-ID-D in a configuration optimized for a large Q_{max} ($0.4 < Q < 25$ Å^{-1}), which is necessary to obtain high resolution in real-space[12]. Glass beads were mounted on Kapton tape in a

transmission geometry, and the intensity of diffracted 100 keV X-rays was measured with a Varex 4343CT area detector at a sample-to-detector distance of ~340 mm. The X-ray beam was approximately 250 μm wide × 100 μm tall, so only a small portion of each bead was measured at a time.

The diffracted intensity from the area detectors was azimuthally integrated with Fit2D[13] and corrected for flat field effects and X-ray polarization. For PDF analysis, scattering data must first be corrected and reduced to obtain the structure factor[12]. Corrections were applied for oblique incidence and detector attenuation[14]. The remaining data reduction was performed with GudrunX software[15]: background subtraction, X-ray fluorescence, sample attenuation, and multiple scattering. Finally, the normalized total scattering structure factor, $S(Q)$, was obtained after subtraction of self and Compton scattering[16] and normalization by the Q-dependent square of the mean X-ray atomic form factor. The "top hat" convolution[17] in GudrunX was applied to eliminate any residual Q-dependent background in $S(Q)$, which otherwise manifests as nonphysical oscillations at r < 1.2 Å in the PDF.

The total structure factor can be expressed as a summation of the atomic partial pair structure factors, s_{ij}, each weighted by its normalized Faber-Ziman weighting factor[18], W_{ij}:

$$S(Q) = \sum_{i,j \geq i} W_{ij} s_{ij}(Q) \tag{S4}$$

$$W_{ij} = \frac{(2 - \delta_{ij}) c_i c_j f_i(Q) f_j(Q)}{\langle f(Q) \rangle^2} \tag{S5}$$

where c_i are the fractional atomic concentrations and $f_i(Q)$ are the X-ray atomic form factors[19]. The differential PDF is given by:

$$D(r) = \frac{2}{\pi} \int_0^{Q_{max}} Q(S(Q) - 1) \sin(Qr)\, dQ \tag{S6}$$

and the total PDF is:

$$T(r) = D(r) + 4\pi \rho r = \sum_{i,j \geq i} W_{ij} \otimes t_{ij}(r) \tag{S7}$$

where ρ is the atomic number density and t_{ij} are the atomic partial pair correlations. A Q_{max} of 24.9 Å^{-1} was used. Densities (Table S2) were extrapolated from glass measurements by Aksay *et al.*[20].

Mean bond distances and coordination for the Si-O and Al-O partials were extracted from the total PDFs by fitting one Gaussian function for each partial to the first peak, using NXFit software[21]. Because the Si-O and Al-O partials overlap substantially, the Si-O coordination was fixed at a value of 4[22], and the Gaussian functions were fit to the data sequentially: first the Si-O contribution was fit using the leading edge of the first peak, then Al-O was fit from the remainder, and finally the two contributions were refined simultaneously with reasonable constraints on the fitting parameters. An example of peak fitting is shown in Fig. S2B for the AS38 glass. From the Si-O and Al-O mean coordination numbers, n_{SiO} and n_{AlO}, the combined O-(Si+Al) coordination was calculated:

$$n_{O-(Si+Al)} \equiv n_{OSi} + n_{OAl} = \frac{c_{Si}}{c_O} n_{SiO} + \frac{c_{Al}}{c_O} n_{AlO} \tag{S8}$$

<u>Structural Modeling</u>

Since all glasses were phase-separated, their structure factors are linear combinations of the endmember phases' structure factors, weighted by composition according to the lever rule. This motivated a linear

regression of the structure factors for AS12, AS23, AS33, AS38, and AS42 to extrapolate and predict the structure factors for the endmembers[23], using estimated compositions of AS7 and AS62.

A structural model for each extrapolated endmember was developed using empirical potential structure refinement (EPSR)[24]. EPSR is a Monte Carlo based technique, in which a simulated volume containing atomic species at the correct concentrations and bulk density is equilibrated using Lennard-Jones and pseudo-Coulomb potentials. An empirical potential is then added that is derived from the disagreement between the modeled structure and experimental measurement. The effect of the empirical potential is to favor Monte Carlo moves that bring the model into closer agreement with the experiment.

The simulations for AS7 and AS62 used 1884 and 2544 atoms, respectively, each in a cube of edge length ~30 Å (ρ = 0.06851 and 0.08673 atoms Å^{-3}, or 2.284 and 2.924 g cm^{-3} [20]). Parameters for the initial atomic potentials are given in Table S3. Each simulation was initially equilibrated at 1000 °C and then at 27 °C. The empirical potential was then activated, first at a magnitude of 10% of the system energy and then at 15%. Once the system stabilized, the model was analyzed to obtain atomic partial pair correlations and coordination number distributions for Si-O and Al-O. Each stage of the simulation (equilibration, empirical potential, and analysis) was run for at least 10,000 iterations.

Atomic configurations were recorded every 10th iteration over 5000 iterations, and these 500 snapshots were further analyzed. Using the R.I.N.G.S. program[25], detailed coordination environments were tabulated for O, and statistics for –(Si/Al)-O- rings were calculated based on King's criterion for shortest path[26]. Bonds for Si-O and Al-O were defined with a cutoff distance of 2.15 Å. Network connectivity was assessed by calculating the fraction of corner-, edge-, and face-sharing connections among (Si/Al)-O_x polyhedra[27]. Visualizations of the glass networks were rendered with CrystalMaker 10.6 software.

Extended Results and Discussion

Bonding and Coordination

For mullite glass, the mean Al-O coordination, n_{AlO}, was obtained by X-ray PDF analysis, ^{27}Al MAS NMR, and EPSR. All three methods yield coordination numbers that agree within their uncertainties: 4.38(7), 4.57(27), and 4.48(5), respectively.

The combined O-(Si+Al) coordination, $n_{O-(Si+Al)}$, increases linearly in AS glasses with Al_2O_3 content (Fig. S2A), which is consistent with all the glasses being phase-separated into varying fractions of the same two endmembers: the atomic structure of the endmembers is the same in all glasses, but their relative contributions to the mean coordination change linearly with bulk composition. This trend in $n_{O-(Si+Al)}$ is also consistent with past studies that have described the Si-rich phase as containing predominantly Al-O_4, while the Al-rich phase contains higher fractions of Al-O_5 and Al-O_6[4,28,29].

Structural Models

Interference functions for the experimental scattering measurements (AS12-AS61) are compared with the extrapolated endmembers (AS7 and AS62) in Fig. S4. Structural models were obtained for the AS7 and AS62 endmember phases via EPSR. These models provide further insights on the atomic partials, coordination environments, and network connectivity than can be obtained from the experimental data alone. The interference functions for the extrapolated experimental data and EPSR are in good agreement (Fig. 4B). Figure S5 shows the total PDFs and weighted atomic partial pair correlations, which illustrate the overlap of the Si-O (green) and Al-O (red) peaks, as well as their very different weighting factors in AS7 (Fig. S5A) vs. AS62 (Fig. S5B) phases. (Weighting factors are provided in Table S4.) The O-O first coordination shell (purple, r ~ 2.7 Å) is broader for the AS62 vs. AS7, resulting in a lack of separation for the total PDF's 2nd and 3rd peaks in AS62. The broader O-O peak in AS62 is reflective of the multiple O coordination environments (Fig. 4D).

Differences in network connectivity for the endmembers were assessed with statistics of -(Si/Al)-O- rings present in the networks. The distribution of ring size is given by R_C in Fig. 4E, which is the number of rings of a given size, normalized by the number of atoms in the simulated volume. The AS7 phase has a modal ring size of 7, in agreement with ring statistics for pure SiO_2 glass[25]. The AS62 phase has a much larger number of rings but with a smaller modal size of 5. The larger number of rings arises partly from the significant fraction of polyhedral edge-sharing, which results in a kind of near-degeneracy: many rings are nearly identical except for tracing a path of connection across one of the two (Si/Al)-O-(Si/Al) linkages present in a given edge-share[25]. This effect has been described previously for a neodymium titanate glass containing 23% edge-sharing of polyhedra[27]. Figure S6 provides the statistic P_N, the fraction of atoms participating in rings of a given size, which provides a better indicator of which ring sizes are most characteristic of a network. Again, AS62 is characterized by smaller rings compared to AS7: for example, AS62 has a P_N maximum of 73% atoms participating for rings containing 4 cations, while AS7 is most characterized by rings containing 5 or 6 cations (P_N = 53 and 43%).

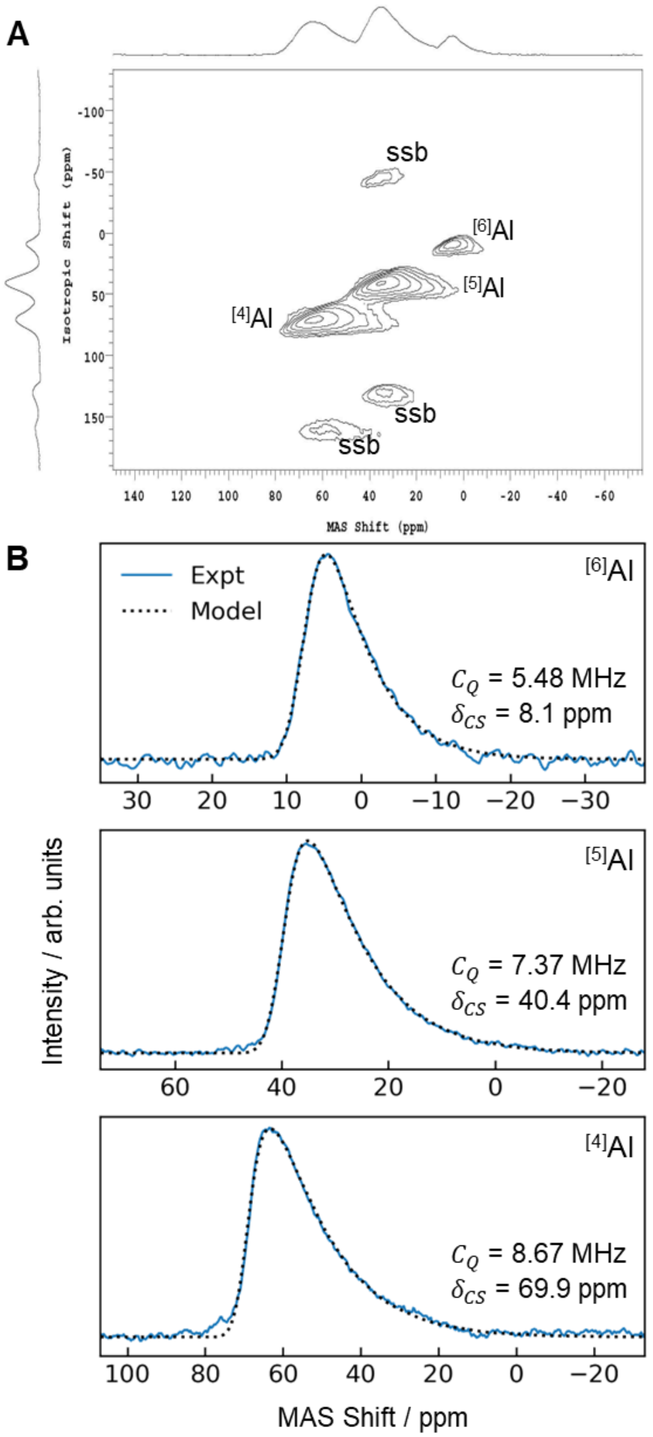

Figure S1 . ^{27}Al 3QMAS NMR spectroscopy of mullite glass. (**A**) Two-dimensional contour plot showing resolution of three distinct Al-O_x peaks and spinning sidebands (ssb). (**B**) MAS slices through the peak maxima in (A) with DMFit analysis of the 2nd-order quadrupolar lineshapes, yielding approximate values of C_Q and δ_{CS}.

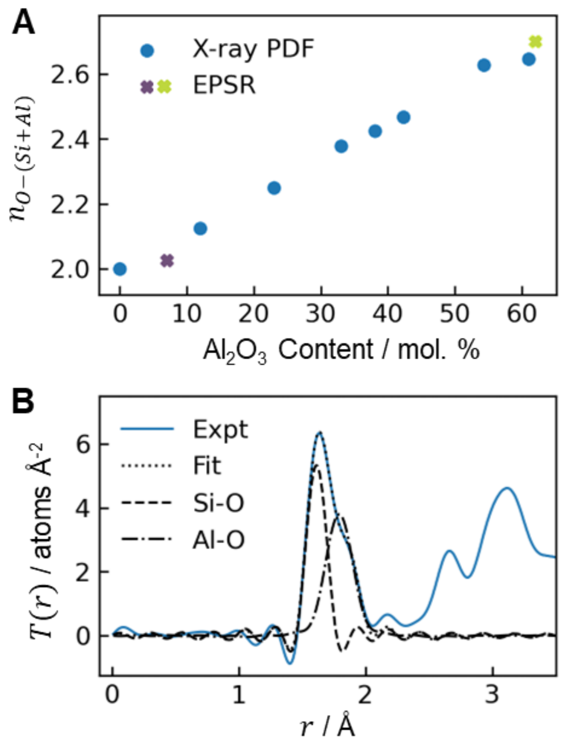

Figure S2. Oxygen coordination in AS glasses. (**A**) Mean oxygen coordination increases linearly with Al_2O_3 content, consistent with glasses' phase separation into AS7 and AS62 endmembers. Uncertainties on X-ray PDF and EPSR values are both ± 0.03. (**B**) Example of Gaussian function fitting to the first PDF peak of AS38 glass, used to extract the mean coordination number for Al-O, assuming that all Si is tetrahedrally coordinated.

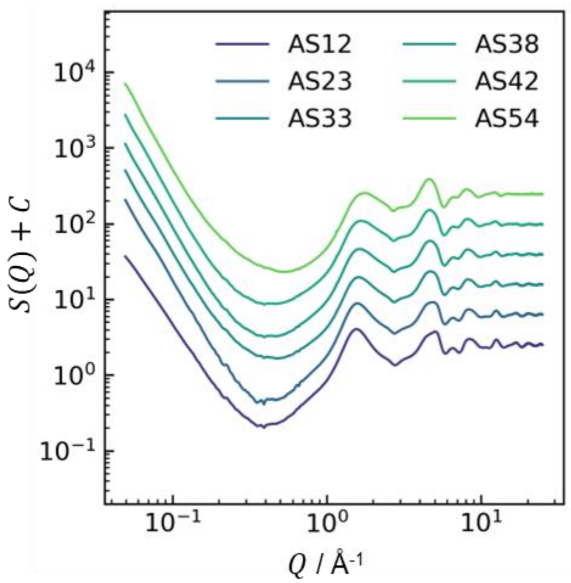

Figure S3. Glasses' structure factors over the small- and wide-angle Q range, showing a Porod slope indicative of phase separation in all glasses. Data for different samples are vertically offset for clarity.

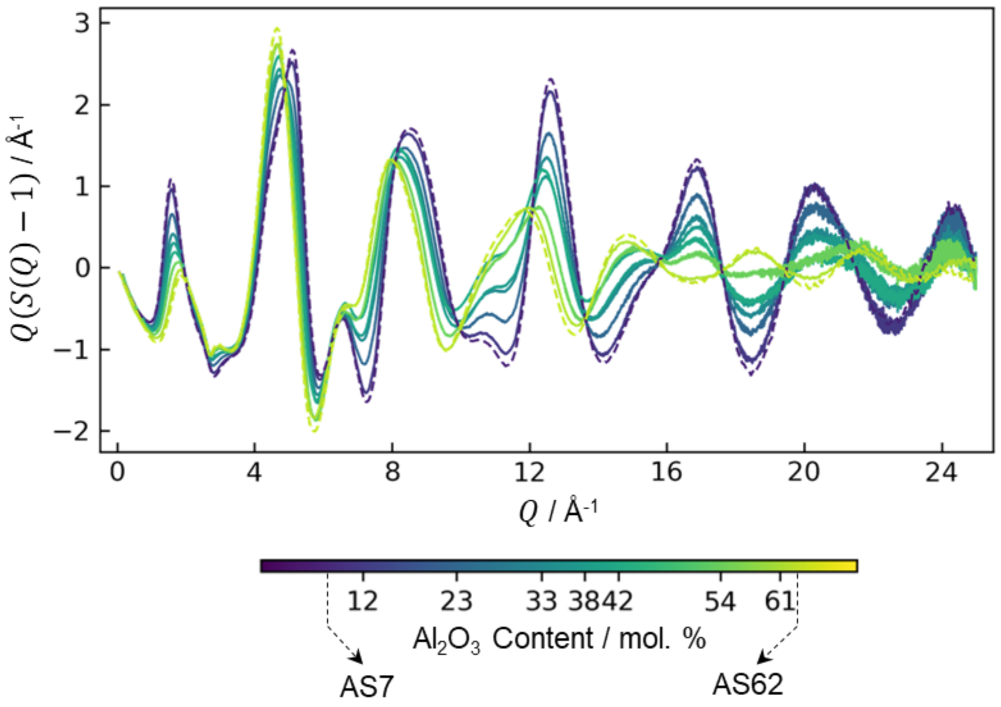

Figure S4. X-ray scattering interference functions for the experimental measurements (AS12-AS61, solid curves), compared with the extrapolated endmembers, AS7 and AS62 (dashed curves).

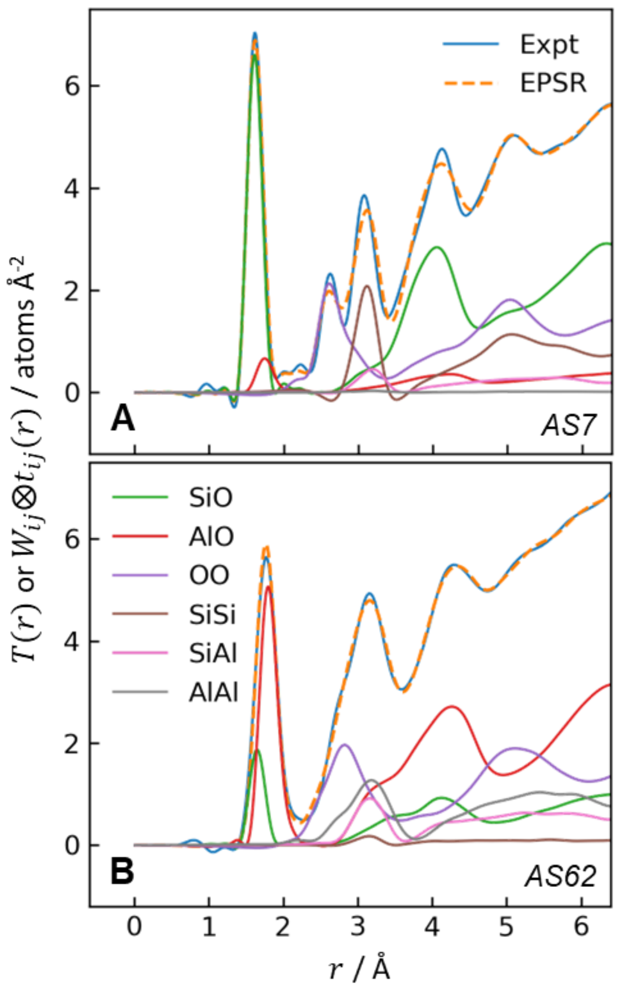

Figure S5. Total PDFs and weighted atomic partial pair correlations from EPSR models for (**A**) AS7 and (**B**) AS62 endmember glasses. A Lorch modification function and Q_{max} of 24.9 Å^{-1} were used for the Fourier transform (see Eqn. S6) for all weighted partials and PDFs.

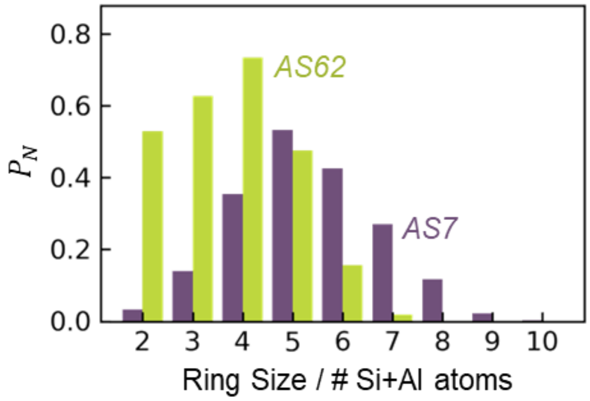

Figure S6. The fraction of atoms participating in -O-Si/Al- rings of a given size, for the AS7 (purple) and AS62 (green) endmember glasses. Standard deviations for several simulation runs are less than ± 0.02.

Table S1. Parameters used to simulate the ^{27}Al MAS NMR spectrum (Fig. 3B).

	δ_{CS} (ppm)	C_Q (MHz)	Population (%)
Al-O_4	67.9(6)	10.2(8)	49.8(4.2)
Al-O_5	40.4(3)	9.0(2)	43.0(4.1)
Al-O_6	9.9(3)	7.1(3)	7.2(1.0)

Table S2. Densities for glasses, interpolated from Aksay *et al.*[20].

**Al_2O_3 (mol. %)**	**Density (g cm^{-3})**
12	2.342
23	2.470
33	2.586
38	2.645
42	2.691
54	2.831
61	2.912

Table S3. Parameters for starting atomic potentials in EPSR[30,31].

**Ion**	**Charge (e)**	**ε (kJ mol^{-1})**	**σ (Å)**
Si^{4+}	+2	0.175	1.03
Al^{3+}	+1.5	0.26	1.26
O^{2-}	-1	0.1625	3.6

Table S4. Faber-Ziman weighting factors (Eqn. S5) for Q = 0 Å^{-1} for the AS glass endmembers.

	**AS7**	**AS62**
W_{SiSi}	0.1719	0.0157
W_{SiAl}	0.0481	0.0954
W_{SiO}	0.4374	0.1241
W_{AlAl}	0.0034	0.1445
W_{AlO}	0.0611	0.3759
W_{OO}	0.2781	0.2444

References for Supplementary Information

1. Weber, J. K. R. The Containerless Synthesis of Glass. *Int. J. Appl. Glas. Sci.* **1**, 248–256 (2010).
2. Risbud, S. H. & Pask, J. A. Calculated Thermodynamic Data and Metastable Immiscibility in the System SiO2-Al2O3. *J. Am. Ceram. Soc.* **60**, 418–424 (1977).
3. Nassau, K., Shiever, J. W. & Krause, J. T. Preparation and Properties of Fused Silica Containing Alumina. *J. Am. Ceram. Soc.* **58**, 461 (1975).
4. Sen, S. & Youngman, R. E. High-resolution multinuclear NMR structural study of binary aluminosilicate and other related glasses. *J. Phys. Chem. B* **108**, 7557–7564 (2004).
5. Massiot, D. *et al.* Modelling one- and two-dimensional solid-state NMR spectra. *Magn. Reson. Chem.* **40**, 70–76 (2002).
6. Neuville, D. R., Cormier, L. & Massiot, D. Al environment in tectosilicate and peraluminous glasses: A 27Al MQ-MAS NMR, Raman, and XANES investigation. *Geochim. Cosmochim. Acta* **68**, 5071–5079 (2004).
7. Kaushik, M. *et al.* Atomic-Scale Structure and Its Impact on Chemical Properties of Aluminum Oxide Layers Prepared by Atomic Layer Deposition on Silica. *Chem. Mater.* **33**, 3335–3348 (2021).
8. Amoureux, J.-P., Huguenard, C., Engelke, F. & Taulelle, F. Unified representation of MQMAS and STMAS NMR of half-integer quadrupolar nuclei. *Chem. Phys. Lett.* **356**, 497–504 (2002).
9. Ilavsky, J. *et al.* Development of combined microstructure and structure characterization facility for in situ and operando studies at the Advanced Photon Source. *J. Appl. Crystallogr.* **51**, 867–882 (2018).
10. Ilavsky, J. Nika: software for two-dimensional data reduction. *J. Appl. Crystallogr.* **45**, 324–328 (2012).
11. Benmore, C. J. *et al.* Extended range X-ray pair distribution functions. *Nucl. Instruments Methods Phys. Res. Sect. A Accel. Spectrometers, Detect. Assoc. Equip.* **955**, 163318 (2020).
12. Benmore, C. J. A Review of High-Energy X-Ray Diffraction from Glasses and Liquids. *ISRN Mater. Sci.* **2012**, 1–19 (2012).
13. Hammersley, A. P. FIT2D: a multi-purpose data reduction, analysis and visualization program. *J. Appl. Crystallogr.* **49**, 646–652 (2016).
14. Skinner, L. B., Benmore, C. J. & Parise, J. B. Area detector corrections for high quality synchrotron X-ray structure factor measurements. *Nucl. Instruments Methods Phys. Res. Sect. A Accel. Spectrometers, Detect. Assoc. Equip.* **662**, 61–70 (2012).
15. Soper, A. K. & Barney, E. R. Extracting the pair distribution function from white-beam X-ray total scattering data. *J. Appl. Crystallogr.* **44**, 714–726 (2011).
16. Balyuzi, H. H. M. Analytic approximation to incoherently scattered X-ray intensities. *Acta Crystallogr. Sect. A* **31**, 600–602 (1975).
17. Soper, A. K. Inelasticity corrections for time-of-flight and fixed wavelength neutron diffraction experiments. *Mol. Phys.* **107**, 1667–1684 (2009).
18. Faber, T. E. & Ziman, J. M. A theory of the electrical properties of liquid metals. *Philos. Mag. A J. Theor. Exp. Appl. Phys.* **11**, 153–173 (1965).
19. Waasmaier, D. & Kirfel, A. New analytical scattering-factor functions for free atoms and ions. *Acta Crystallogr. Sect. A* **51**, 416–431 (1995).
20. Aksay, I. A., Pask, J. A. & Davis, R. F. Densities of SiO2-Al2O3 Melts. *J. Am. Ceram. Soc.* **62**, 332–336 (1979).
21. Pickup, D., Moss, R. & Newport, R. NXFit: A program for simultaneously fitting X-ray and neutron diffraction pair-distribution functions to provide optimized structural parameters. *J. Appl. Crystallogr.* **47**, 1790–1796 (2014).
22. Benmore, C. J. *et al.* Structural and topological changes in silica glass at pressure. *Phys. Rev. B* **81**, 54105 (2010).
23. Alan K. Soper & Maria Antonietta Ricci. Structures of High-Density and Low-Density Water. *Phys. Rev. Lett.* **84**, 2881 (2000).
24. Soper, A. K. Empirical potential Monte Carlo simulation of fluid structure. *Chem. Phys.* **202**, 295–

306 (1996).
25. Le Roux, S. & Jund, P. Ring statistics analysis of topological networks: New approach and application to amorphous GeS2 and SiO2 systems. *Comput. Mater. Sci.* **49**, 70–83 (2010).
26. King, S. V. Ring Configurations in a Random Network Model of Vitreous Silica. *Nature* **213**, 1112–1113 (1967).
27. Wilke, S. K., Alderman, O. L. G., Benmore, C. J., Neuefeind, J. & Weber, R. Octahedral oxide glass network in ambient pressure neodymium titanate. *Sci. Rep.* **12**, 8258 (2022).
28. McMillan, P. & Piriou, B. The structures and vibrational spectra of crystals and glasses in the silica-alumina system. *J. Non. Cryst. Solids* **53**, 279–298 (1982).
29. Hudon, P. & Baker, D. R. The nature of phase separation in binary oxide melts and glasses. I. Silicate systems. *J. Non. Cryst. Solids* **303**, 299–345 (2002).
30. Bowron, D. T. Building Monte Carlo Models of Glasses Using Neutron and/or X-ray Diffraction Data. *Procedia Mater. Sci.* **7**, 38–52 (2014).
31. Weigel, C., Cormier, L., Calas, G., Galoisy, L. & Bowron, D. T. Intermediate-range order in the silicate network glasses NaFexAl1-xSi2O6 (x=0,0.5,0.8,1): A neutron diffraction and empirical potential structure refinement modeling investigation. *Phys. Rev. B - Condens. Matter Mater. Phys.* **78**, 1–11 (2008).
